# Development of transgenic *Daphnia magna* for visualizing homology-directed repair of DNA

**DOI:** 10.1038/s41598-022-06526-8

**Published:** 2022-02-15

**Authors:** Rizky Mutiara Fatimah, Nikko Adhitama, Yasuhiko Kato, Hajime Watanabe

**Affiliations:** 1 Department of Biotechnology, Graduate School of Engineering, Osaka University, Suita, Osaka Japan; 2 Biotechnology Global Human Resource Development Program, Division of Advanced Science and Biotechnology, Department of Biotechnology, Graduate School of Engineering, Osaka University, Suita, Osaka Japan

**Keywords:** Homologous recombination, Model invertebrates

## Abstract

In the crustacean *Daphnia magna*, studying homology-directed repair (HDR) is important to understand genome maintenance during parthenogenesis, effects of environmental toxicants on the genome, and improvement of HDR-mediated genome editing. Here we developed a transgenic *D. magna* that expresses green fluorescence protein (GFP) upon HDR occurrence. We utilized the previously established reporter plasmid named DR-GFP that has a mutated *eGFP* gene (*SceGFP*) and the tandemly located donor *GFP* gene fragment (*iGFP*). Upon double-strand break (DSB) introduction on *SceGFP*, the *iGFP* gene fragment acts as the HDR template and restores functional eGFP expression. We customized this reporter plasmid to allow bicistronic expression of the *mCherry* gene under the control of the *D. magna EF1α-1* promoter/enhancer. By CRISPR/Cas-mediated knock-in of this plasmid via non-homologous joining, we generated the transgenic *D. magna* that expresses mCherry ubiquitously, suggesting that the DR-GFP reporter gene is expressed in most cells. Introducing DSB on the *SceGFP* resulted in eGFP expression and this HDR event could be detected by fluorescence, genomic PCR, and quantitative reverse-transcription PCR, suggesting this line could be used for evaluating HDR. The established reporter line might expand our understanding of the HDR mechanism and also improve the HDR-based gene-editing system in this species*.*

## Introduction

Genomes are threatened by endogenously generated chemicals like reactive oxygen species and exogenous compounds such as mutagenic agents and radiation^1^, which can lead to DNA double-strand breaks (DSBs). To ensure genetic stability and cellular viability, repairing the DSBs is essential. The DNA repair mainly occurs through non-homologous end joining (NHEJ) and homology-directed repair (HDR)^2^. The NHEJ leads to ligation of the two ends of the DNA strand during which insertion or deletion of nucleotides (indels) can often occur at the cleavage site. The HDR repairs the DSBs by using information copied from undamaged DNA that has an identical or homologous sequence (homology)^3^ This homology-directed repair system can be divided into four sub-pathways based on the mechanistic difference: double-strand break repair (DSBR), synthesis-dependent strand annealing (SDSA), break-induced replication (BIR), and single-strand annealing (SSA)^2^. First, in the DSBR, the formation of an intermediate structure with the Holliday junctions (HJs) leads to the generation of crossover and non-crossover products^4^. Second, the SDSA exclusively generates the non-crossover products due to the lack of formation of the HJ structure^5^. Third, when only one ended DSB site has a sequence similar to that of the template, BIR occurs for non-reciprocal translocation of genetic information from the template strand^6^. Fourth, when the DSB is induced between the tandem repeats of the highly homologous regions, the SSA repairs the DNA by pairing the homologous region followed by deletion of unpaired DNA and the intervening region^7^. In the mitotically proliferating cells, the SDSA is known to be the most common among the HDR sub-pathways^8^.

The HDR also plays an important role in the field of genome editing due to its nature of high fidelity and accuracy. Unlike NHEJ, HDR avoids multiple integrations of a donor DNA and indels between transgene and surrounding genomic region. Thus, following DSB using the programmable nuclease for instance TALEN or CRISPR-Cas, a precise genome modification such as the codon replacements or the seamless integration of the fluorescent reporter gene can be achieved by using a donor DNA flanked with right and left locus-specific homology arms as a template^9,10^.

The water flea *Daphnia magna* is a small freshwater crustacean found in broad continents such as Europe, the Middle East, Central Asia, Africa, and North America^11^*.* The genus *Daphnia* reproduces by parthenogenesis under favorable environmental conditions but switches it to sexual reproduction in response to environmental stimuli such as shortened photoperiod, a lack of food, and/or increased population density^12^. The sequenced genome of *Daphnia* reveals highly duplicated genes, resulting in tandem gene clusters^13^*.* These tandem clusters may serve as a template for HDR-based repair to attenuate the effect of deleterious mutations during the parthenogenetic cycle, which suggests that *Daphnia* may have a unique HDR mechanism.

*Daphnia magna* occupies an important position in the freshwater food chain and is highly sensitive to chemicals, which makes this species a model in environmental and toxicological studies. The effects of genotoxicants have been investigated at the phenotypic level^14,15^. To understand their actions at the molecular level, it is important to study the DNA repair mechanism in this species. In the field of genome editing, the HDR-based knock-in of the exogenous DNA fragments has been reported in *D. magna*^16^ as well as NHEJ-mediated knock-in^17,18^. The HDR-based knock-in efficiency was low probably due to competition with the NHEJ pathway. To test this hypothesis, disruption *DNA ligase IV* which is the conserved component of the NHEJ pathway has been attempted^16^ However, its effect was not fully evaluated due to the lack of a method for quantifying the HDR event in vivo. Therefore, a system to evaluate and quantify the HDR event is a necessity.

**Figure 1:**
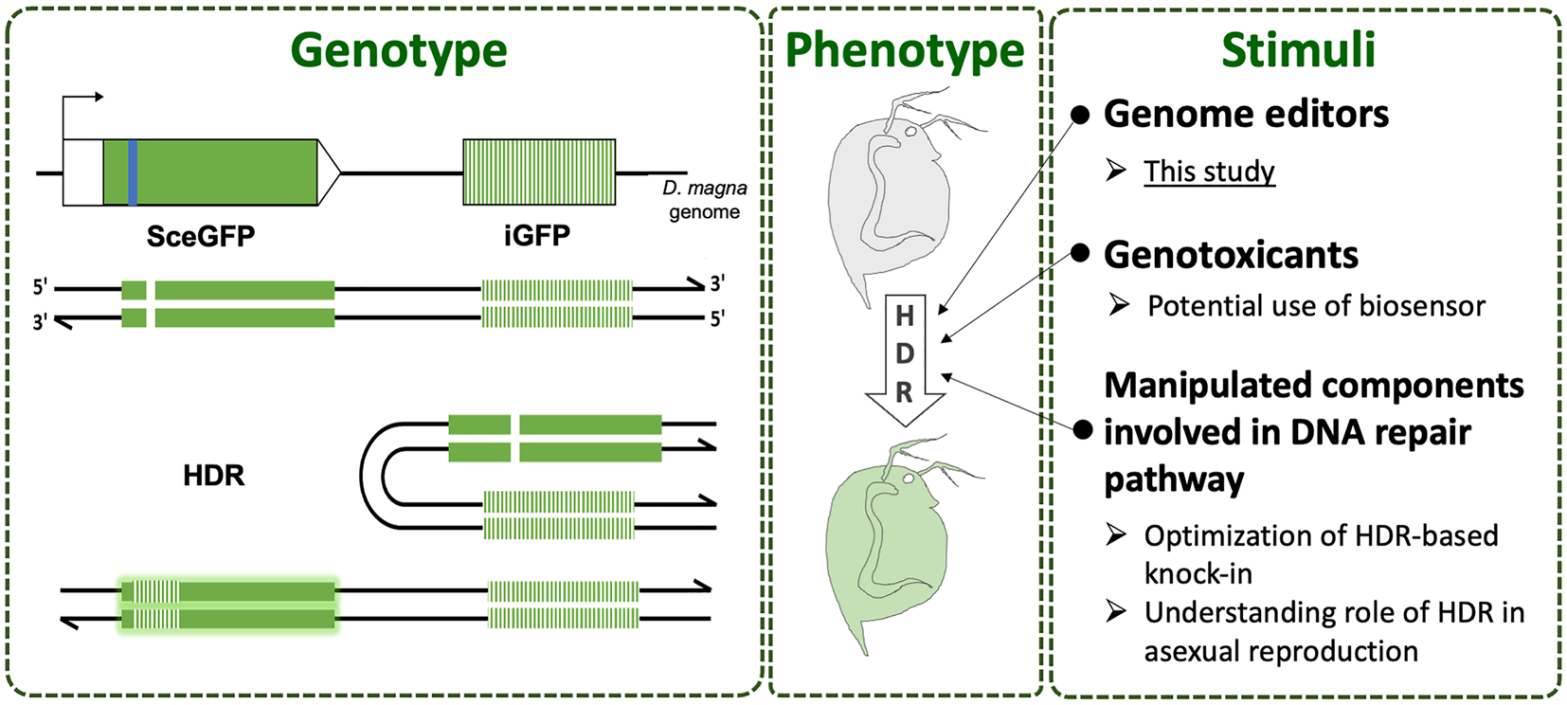
The DR-GFP reporter assay and its potential application. The DR-GFP reporter consists of two tandem repeats of mutated GFP. The first repeat (SceGFP, solid green) contains an I-SceI recognition site with stop codons embedded (blue line). The second repeat (iGFP, striped-green) lacks 5′and 3′sequence. The introduction of DSB at the I-SceI site induces HDR-based DNA repair, utilizing homologous sequences from the iGFP, which in turn resulted in a functional *eGFP* gene (left box figure). The eGFP fluorescence signal will be observed in *D. magna* transgenic reporter as a result of the HDR event (middlebox figure). The possible application of the transgenic reporter is to elucidate the effect of various stimuli on the HDR level (right box).

Fluorescence live imaging of the HDR event is essential not only for investigating how and where the genome integrity is maintained in living organisms but also for evaluating the HDR activity by manipulating the components for DNA repair. The direct repeat GFP (DR-GFP) reporter assay has been established for fluorescence-based visualization of the HDR activity^19^. The DR-GFP reporter system is composed of two mutated eGFP genes (Fig. 1, Genotype). The upstream eGFP gene named *SceGFP* contains a recognition site of the rare-cutting I-SceI restriction enzyme. This recognition site contains two in-frame stop codons to terminate the protein expression. At downstream of the *SceGFP*, there is another mutated *eGFP* fragment termed internal GFP or *iGFP* that is an 812-bp internal GFP fragment. The HDR event can be detected by introducing a double-strand break (DSB) with I-SceI in the inactive *SceGFP* gene. The cleavage site will be repaired by HDR using *iGFP* as the template. Among the HDR sub-pathways, this DR-GFP system can visualize the non-crossover events that are mediated by the DSBR and SDSA^20^, suggesting that this reporter can visualize the major HDR events spatiotemporally in vivo. This reporter has been applied to study the factors that contribute to HDR in mouse^21^ and to study the role of a transcriptional repressor protein in HDR using *C. elegans* models^22^.

Here we integrated the DR-GFP reporter system in the *D. magna* genome (Fig. 1, Genotype). We confirmed its functionality by introducing DSBs at the *SceGFP* region with the CRISPR/Cas9 system and detecting the eGFP signal spatiotemporally (Fig. 1, Phenotype). Furthermore, we could detect the repaired eGFP gene by genomic PCR and qPCR, which adds merit to this system to be utilized for the evaluation of the HDR event. By applying different stimuli (Fig. 1, Stimuli), the established transgenic *Daphnia* might contribute to various scientific fields such as ecotoxicology, genome editing, and evolutionary biology.

## Materials and methods

### Daphnia* magna* strain and culture condition

Wild type *D. magna* (NIES clone) was obtained from the National Institute of Environmental Studies (NIES, Tsukuba, Japan) and has been maintained in the laboratory for many generations. The *D. magna* was cultured under the following conditions: 80 juveniles (less than 24 h old) were collected and cultured in 5 L Artificial *Daphnia* Medium (ADaM)^23^ at temperature 22–24 °C, under 16 h/8 h of light/dark photoperiod. *D. magna* were fed daily with 8 × 10^9^ cells of *Chlorella vulgaris* (Oita Medaka Biyori, Oita, Japan) and 3 mg of baker’s yeast (Marusan Pantry, Ehime, Japan) during the first week. Later, juveniles were removed daily and amounts of chlorella and yeast extract were doubled. The culture medium was changed once a week.

### Customization of reporter plasmid

**Figure 2:**
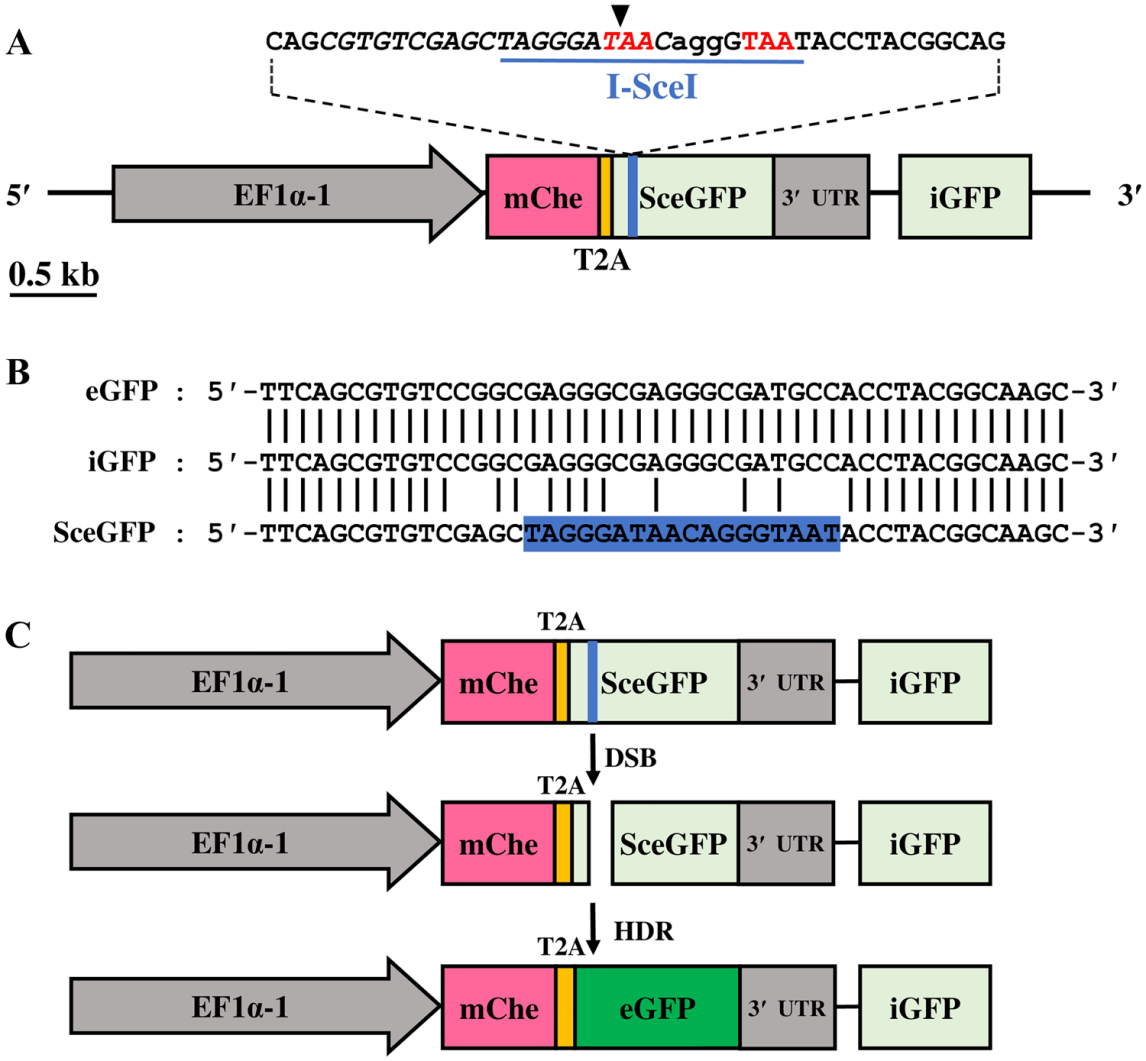
DR-GFP reporter system. (**A**) The donor plasmid design. The direct repeat of differentially mutated *eGFP* (DR-GFP) consists of mutated (*SceGFP*) and 5′ and 3′-lacking sequence of *eGFP* (*iGFP*), both are indicated in light green boxes. Three repeats (two complete and one partial) of the simian virus 40 large T-antigen nuclear localization signal (SV40 NLS) are included in the *SceGFP* sequence (Supplementary Figure S1). The reporter system is expressed ubiquitously under *D. magna EF1α-1* promoter/enhancer (grey arrow). The red fluorescence protein mCherry-coding sequence is placed upstream of the DR-GFP system. *mCherry* and DR-GFP are bicistronically expressed using *Thosea* *asigna* virus 2A (T2A) peptide indicated in the yellow box. *SceGFP* contains a recognition site of the 18 bp I-SceI restriction enzyme and in-frame two stop codons indicated in the blue underline and red letter respectively. SceI gRNA was designed to correspond with the I-SceI recognition site (italic) upstream of the PAM sequence (small letter). The cleavage site of SceI gRNA was indicated by a black triangle. (**B**) The alignment between eGFP, iGFP, and SceGFP sequences. Blue areas indicate the I-SceI site. (**C**) The diagram of the DR-GFP system for reporting HDR events. Double-strand break (DSB) is introduced in the I-SceI site by the Cas9-gRNA complex. Following homology-directed repair (HDR) occurrence, *iGFP* will serve as a repair template, leading to *SceGFP* repair indicated by eGFP expression green box.

To visualize the HDR by fluorescence in *D. magna*, a reporter donor plasmid pEF1α-1::mCherry-2A-DR-GFP was constructed by customizing the previously established pDRGFP plasmid (Addgene No.26475)^19^ (Fig. 2A, B). To allow ubiquitous expression in *D. magna*, the original chicken *β-actin* promoter was replaced with a 2.3 kb of *D. magna* elongation factor 1 *α-1* (*EF1α-1*) promoter/enhancer, including the transcription start site, the complete first intron, and part of the second exon with a start codon^17,24^. In addition, to recapitulate *EF1α-1* endogenous expression, a full-length *EF1α-1* 3′ UTR was added downstream of the reporter. We retained the original two mutated eGFP fragments (*SceGFP* and *iGFP*) along with their nuclear localization sequence to distinguish the eGFP-expressing cells individually.

DR-GFP reporter will function when the DSB is introduced in the I-SceI site. By SDSA or non-crossover DSBR subpathway of HDR, *SceGFP* will use *iGFP* as a repair template resulting in the functional eGFP expression (Fig. 2C). A red fluorescent protein gene *mCherry* ORF was fused upstream of the *SceGFP* via a sequence encoding *Thosea asigna* virus 2A (T2A), which can lead to bicistronic expression of both *mCherry* and mutated/repaired *eGFP*^25^. Lastly, for the integration into *D. magna* genome, a 200 bp sequence of *scarlet* gene harboring a gRNA target sequence^26,27^ was added. The complete nucleotide sequence of the customized DR-GFP reporter and the deduced amino sequence is provided in Supplementary Figure S1. All assemblies were performed using GeneArt Cloning & Assembly (Invitrogen, Carlsbad, USA). The constructed donor plasmid was purified using FastGene Plasmid Mini Kit (Nippon Genetics, Tokyo, Japan) and sequenced. The donor plasmid used for microinjection was purified using PureYield Miniprep (Promega, Madison, USA) followed by phenol–chloroform purification, two times ethanol washing, and was re-suspended with ultrapure water (Invitrogen).

### In vitro RNA synthesis

Guide RNAs (gRNAs) were synthesized using a cloning-free method from PCR-amplified template DNA as previously described^27^. The sense synthetic oligonucleotide containing a T7 promoter sequence, a gene-specific target sequence, and the first 20 nt of the Cas9 binding scaffold are shown in Table 1. gRNAs were synthesized using the MegaScript T7 Transcription Kit (Invitrogen), purified using Roche Mini Quick Spin RNA Column (Roche, Mannheim, Germany) followed by phenol/chloroform extraction, ethanol precipitation.

**Table 1:** The sense sequence of the oligonucleotide for guide RNA synthesis.

No	gRNA target	Sense oligonucleotide
1	Scarlet (st)	5′-GAAATTAATACGACTCACTATA GGTTCACTCGTCGCCTTAAT*GTTTTAGAGCTAGAAA*-3′
2	Distal-less (Dll)	5′-GAAATTAATACGACTCACTATA GCAAGAAGATGCGCAAACCG*GTTTTAGAGCTAGAA*-3′
3	SceI	5′-GAAATTAATACGACTCACTATA GGTGTCGAGCTAGGGATAAC*GTTTTAGAGCTAGAA*-3′

A T7 promoter, a targeting sequence, and the first 20 bp of the Cas9 binding scaffold sequence were indicated with bold letters, underline, and italic letters respectively.

For Cas9 mRNA synthesis, a template DNA containing T7 promoter sequence was PCR amplified from pCS-Dmavas-Cas9^28^. Capped mRNA synthesis and poly(A) tail addition were performed using mMessage mMachine T7 kit and Poly(A) Tailing Kit (Invitrogen) respectively. Synthesized mRNA was column purified, followed by phenol/chloroform extraction and ethanol precipitation. mRNA integrity and the addition of poly(A) tails were confirmed by denaturing formaldehyde gel electrophoresis.

### Generation of HDR reporter transgenic *Daphnia magna*

For the generation of the DR-GFP line, the customized DR-GFP reporter plasmid was integrated into *D. magna* genome by utilizing the CRISPR/Cas-mediated knock-in via non-homologous end-joining^17^. Microinjection into *Daphnia* embryos was performed following an established protocol using the *S. pyrogenes*-originated Cas9 proteins^17,29^. The Cas9 proteins were expressed in *E. coli* strain BL21 (DE3) and purified following established protocol^30^. Fifty nanograms per microliter of purified donor plasmid was co-injected with 2 µM *scarlet* targeting gRNA, and 1 µM Cas9 protein. Shortly before the microinjection, Cas9 protein and gRNA were incubated at 37 °C for 5 min to form a ribonucleoprotein (RNP) complex. Microinjection was performed within two hours after the preparation of the solution. After injection, the intact eggs were transferred, cultured individually in a sterile 96 well plate, and put in an incubator at 22 °C with 16 h/8 h of light/dark photoperiod for 3 days. Each well of the 96 well-plate was filled with 100 μL of M4-sucrose. Transgenic candidates were screened based on the mCherry expression in the ovary of injected embryos (G0). mCherry expressing offspring were cultured and genotyping was performed using the second generation offsprings (G2).

### Genotyping

*Daphniids* were collected and homogenized in 500 µL lysis buffer (50 mM Tris-HCl pH 7.5, 20 mM EDTA pH 8.0, 100 mM NaCl, 1% SDS) using MicroSmash homogenizer (TOMY, Tokyo, Japan) at 3000 rpm for 1.5 min with the presence of 0.15 mg/mL Proteinase K (Nacalai Tesque, Kyoto, Japan). The homogenized *daphniids* (lysate) were shaken overnight at 55 °C, 450 rpm using an incubator shaker (Bioshaker M-BR-022UP, TAITEC, Tokyo, Japan). To obtain genomic DNA (gDNA), the lysate was purified using phenol/chloroform extraction, precipitated with isopropanol, rinsed twice with 70% ethanol, and dissolved in 50 µL TE buffer before being used as a template for genomic PCR. The PCR was performed by using an Ex Taq Hot-Start DNA polymerase (TaKaRa) with primer sets amplifying the target region as described in Table 2.

**Table 2:** List of primers.

Purpose	Target region	Direction	Sequence
Genotyping	mCherry	Forward	5′-GGCCATCATCAAGGAGTTC-3′
		Reverse	5′-CGTTGTGGGAGGTGATGTC-3′
	5′ junction region of integration site	Forward	5′-TGGAGACGTCATTCGATTACG-3′
		Reverse	5′-CTGGCGTAATAGCGAAGAGG-3′
	3′ junction region of integration site	Forward	5′-CAGCCATACCACATTTGTAG-3′
		Reverse	5′- GTTGAGCGACTGGTATCTTC -3′
	Repaired eGFP	Forward	5′-CCAGACCGCCAAGCTG AAGGTGACC-3′
		Reverse	5′-ATCGCCCTCGCCCTCGCCG-3′
qPCR	Repaired eGFP	Forward	5′-TTCTAACATGCGGGGACGTG-3′
		Reverse	5′-CAGCTTGCCGTAGGTGGCAT-3′
	mCherry	Forward	5′-CTACGACGCTGAGGTCAAGAC-3′
		Reverse	5′-GGTGTAGTCCTCGTTGTGGG-3′
	L32	Forward	5′-GACCAAAGGGTATTGACAACAGA-3′
		Reverse	5′-CCAACTTTTGGCATAAGGTACTG-3′

The primers were synthesized by FASMAC (Tokyo, Japan).

### Functional analysis of DR-GFP reporter transgenic *D. magna*

To demonstrate the functionality of the DSB reporter in *Daphnia*, we designed a gRNA named SceI gRNA that introduced DSB at 2 bp upstream of the I-SceI digestion site that was previously used for I-SceI-dependent DSBs^19^.

To confirm whether Cas9 was active during microinjection, we also co-injected the SceI gRNA with another gRNA targeting *Distal-less (Dll)* gene. Previously RNAi-mediated knockdown of *Dll* in embryos of *D. magna* led to a distinct phenotype “truncation of second antennae” and the level of this truncation corresponded to the degree of impairment of this gene^29^. The Dll gRNA was designed to target the upstream of the homeodomain region in exon 2 (Supplementary Figure S2), as this region is highly conserved among arthropod^29^ and considered important for Dll function^31,32^.

The phenotypes of the second antennae of the injected embryos were observed 48 h post-injection (hpi) and categorized as normal, mild, medium, or strong truncation following the previous study. In normal phenotype, second antennae consist of a protopodite (*P*), carrying a dorsal and ventral ramus. Each ramus has three segments, Terminal (*T*), Middle (*M*), and Basal (*B*). There is an additional small wedge-shaped segment (*w*) between *B* and *P*. Mild truncation exhibit, a part of *M* and full *B* (ventral ramus), full *M*, *B*, and *w* (dorsal ramus). Medium truncation: a trace of *B* (ventral), *B,* and *w* (dorsal). Strong truncation: only a trace of *B* and *w* (dorsal)^29^.

### Fluorescence photography

Fluorescence images were photographed using the Leica DC500 CCD Digital Camera mounted on the Leica M165FC fluorescence microscope (Leica Microsystem, Wetzlar, Germany). The mCherry filter (Leica Microsystem) was used to screen the DR-GFP line. The GFP3 filter (Leica Microsystem) was used to observe repaired *SceGFP* after the HDR.

### Total RNA extraction

Injected and uninjected embryos were collected at 48 h post-injection. Injected embryos were sorted based on the presence of eGFP expression and its total RNA was extracted in the presence of Sepasol-RNA I solution (Nacalai Tesque) according to the manufacturer’s protocol. The total RNA was purified using phenol/chloroform extraction, precipitated with 70% ethanol, dissolved in UltraPure water (Invitrogen), and directly subjected to reverse transcriptase reaction.

### Quantitative reverse-transcription PCR (qPCR)

cDNAs were synthesized from 1 µg of purified total RNA using random primers and PrimeScript II 1st strand cDNA Synthesis Kit (TaKaRa). The *β-actin* gene was amplified by PCR to confirm the absence of genomic DNA contamination as previously described^33^. To quantify repaired *SceGFP* mRNA level, qPCR was performed in StepOnePlus (Applied Biosystem) using KOD SYBR® qPCR Mix (Toyobo, Osaka, Japan) with primers as listed in Table 2. The cycling condition is as follows: 2 min at 98 °C, followed by a total of 40 cycles of 98 °C for 10 s, 60 °C for 10 s, and 68 °C for 30 s. Primers’ specificity was confirmed by melting curve analysis and agarose gel electrophoresis. The expression level of *eGFP* was normalized using that of *mCherry* that is controlled under the same promoter.

## Results

### The genotype of HDR reporter transgenic *Daphnia*

**Figure 3:**
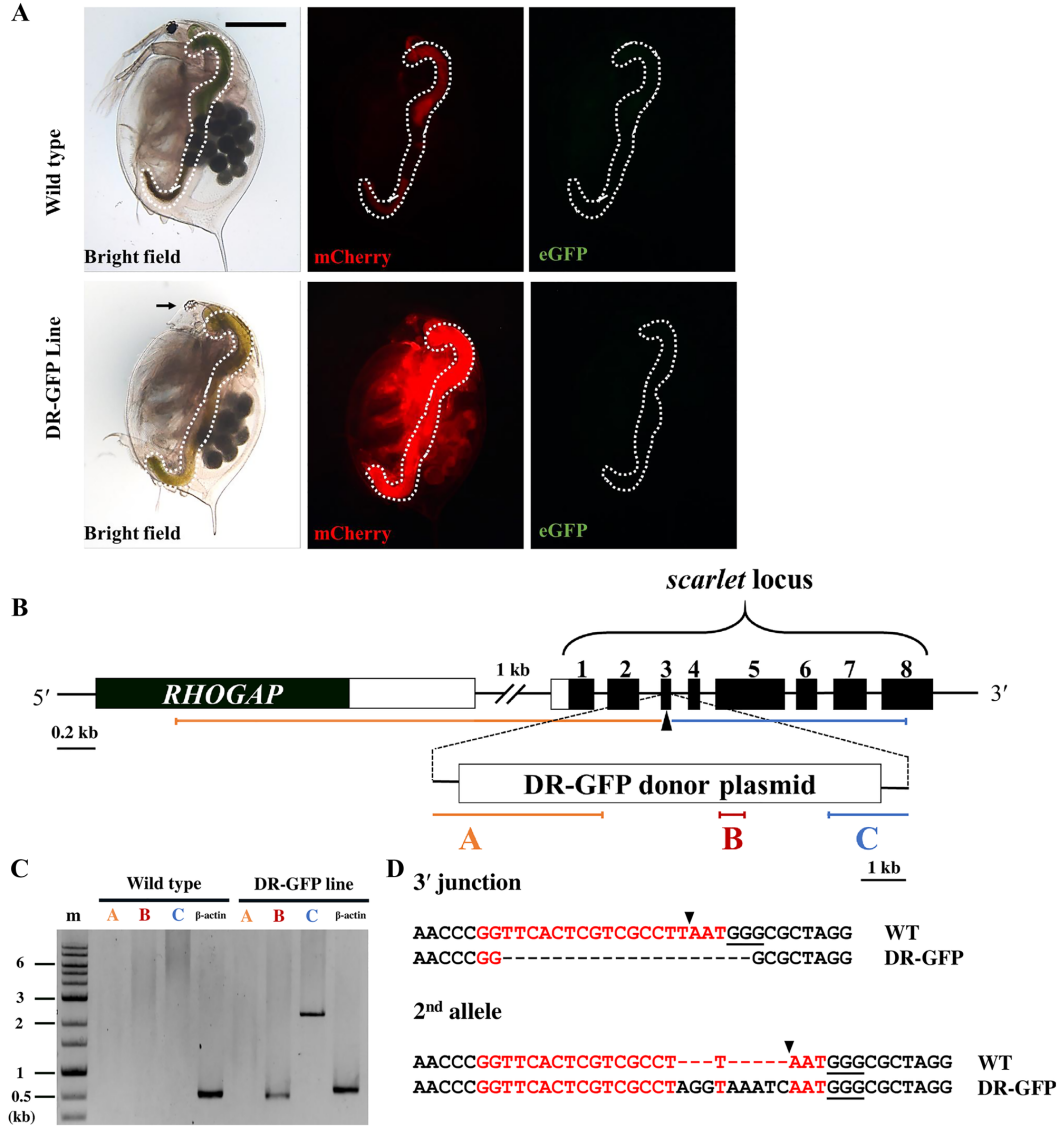
The phenotype and genotype of the DR-GFP line (**A**) Comparing phenotypes of the DR-GFP line with the wild type *Daphnia*. The top and lower rows show *D. magna* obtained from wild type and DR-GFP lines respectively. The image in each column was photographed using either of bright field, mCherry, or GFP3 filter. The region inside the white-dashed line is the gut, in which ingested chlorella emits slight red autofluorescence both in wild type and DR-GFP. Widespread mCherry fluorescence was observed only in the transgenic line, while eGFP fluorescence was not observed. Black arrow indicates loss of black eye pigment due to disrupted *scarlet* allele. (**B**) Schematic representation of the integration site of the DR-GFP donor plasmid. A part of the *RHOGAP* (Rho-GTPase activating protein) gene is shown upstream of *scarlet*. The DR-GFP donor plasmid was integrated into exon 3 of the *scarlet* gene. A and C indicate the 5′ and 3′ junction regions of donor plasmid and genome, B indicated the internal region of donor plasmid (*mCherry* gene). (**C**) PCR result was visualized by gel electrophoresis. The first lane is the marker, followed by fragments A, B, C, and β-actin (beta-actin) for both wild type and DR-GFP line *Daphnia*. In the sample using DR-GFP line *Daphnia,* all fragments except the 5′ junctions were amplified. The full length of the gel is presented in Supplementary Figure S4 (**D**) 20 bp deletion and 8 bp insertion were detected in the 3′ junction of plasmid integration and another allele of *scarlet* respectively. The red-colored nucleotides and black triangle indicated the St gRNA target and DSB site respectively.

To establish the HDR reporter transgenic *Daphnia* (DR-GFP line), we co-injected reporter plasmid and the RNP complex into 29 eggs. The 10 injected embryos survived until adult, from which 9 produced offspring with a white eye that is the typical phenotype of the *scarlet* mutant, indicating that the Cas9 RNP induced DSBs at the targeted site*.* Of the 9, one adult produced offspring with ubiquitous mCherry fluorescence, suggesting germline transmission of reporter plasmid (Fig. 3A). This fluorescence pattern also indicates that this reporter system enables us to detect the HDR event in most types of cells. We cultured this potentially transgenic line for genotyping.

To investigate whether NHEJ-mediated knock-in occurred, genotyping was performed using the genome of the potentially transgenic line. We amplified the mCherry fragment, 5′ and 3′ junctions between the transgene and its surrounding region by PCR (Fig. 3B). The expected size of the mCherry fragments was obtained only in the potential transgenic line (Fig. 3C, fragment B, DR-GFP line). The 3′ junction region was also amplified in this line by using forward primer targeted at the downstream of *EF1α-1* 3′ UTR of the donor plasmid and reverse primer targeted exon 8 of *scarlet* gene locus (Fig. 3B, C, fragment C, DR-GFP line). Sequencing of this PCR product confirmed the integration of the reporter plasmid at the *scarlet* locus and revealed 20 bp deletion and 8 bp insertion at the 3′ side of the integrated cassette (Fig. 3D, 3′ junction). Consistent with the white-eyed phenotype, another allele contained indel mutation at the DSB site (Fig. 3D, 2nd allele). We were unable to amplify the 5′ junction region even if the forward primer was designed at 3157 bp upstream and 2610 bp downstream of the DSB site (Fig. 3B, C, fragment A, DR-GFP line). This suggests that large deletion occurred at the 5′ side of the integration site. Nevertheless, amplification and sequencing of the full-length of the DR-GFP gene cassette demonstrated the integration of the intact DR-GFP reporter (Supplementary Figure S1).

### The DSB near the I-SceI site leads to the generation of eGFP-positive cells in embryos of the DR-GFP line

**Figure 4:**
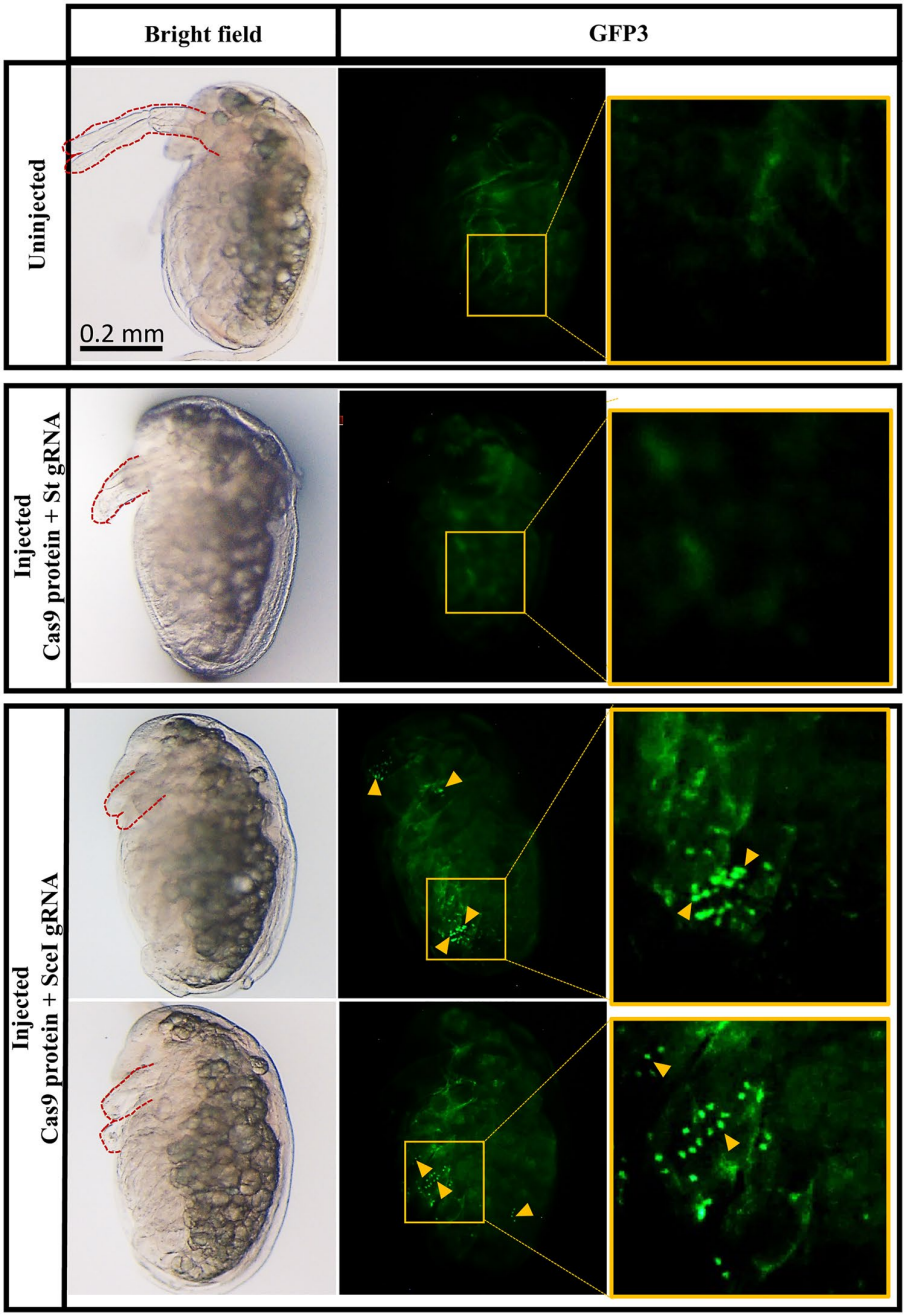
Detection of eGFP-positive cells of DR-GFP transgenic *D. magna* following the DSB of the *SceGFP*. DR-GFP line was co-injected with Cas9 protein, SceI gRNA, and Dll gRNA. The injection of Cas9 protein with St gRNA and Dll gRNA was performed as a control. The first row shows uninjected control, while the second, third, and fourth rows show injected individually *Daphnia*. Images were taken using the bright field and the GFP3 filter. All daphniids were photographed at 48 h post-injection. The red dashed line shows the second antennae region, which was truncated because of Dll gRNA injection. The weak background green fluorescence observed throughout the body of all samples was coming from autofluorescence. The repaired *SceGFP* was controlled under *EF1α-1* promoter/enhancer and contains NLS, resulting in abundantly expressed and nuclear-localized eGFP expression (yellow triangles).

To examine whether the DR-GFP reporter gene is functional in the established line, we attempted to introduce the DSB near the I-SceI site. Seventy-four eggs were co-injected with 1 µM Cas9 protein and gRNA mixtures (SceI gRNA and Dll gRNA, 2 µM each). Dll gRNA was used as a marker for Cas9 activity during microinjection as described in Materials and methods and Supplementary Table S1. Forty-three embryos survived until the 48 hpi stage and 41 (95%) showed truncation of the second antennae (Table 3) from which, 22 embryos (54%) showed the strong phenotype^29^, indicating that Cas9 was active during injection and could introduce DSBs on the genome. Of the 41, 33 (80%) showed strong nuclear-localized eGFP fluorescence in the tissues such as the head and thoracic appendages (Fig. 4). In contrast, embryos injected with Cas9 RNP including the unrelated *St* gRNA (Fig. 4) and Dll gRNA did not show intense and nuclear-localized GFP signal, indicating that the recovery of the eGFP fluorescence occurred by injection of Cas9 protein and SceI gRNA.

**Table 3:** Summary of Cas9 protein, SceI gRNA, and Dll gRNA co-injection.

Injected	Developed (48hpi)	Truncated antennae			Nuclear-localized eGFP
		Strong	Medium	Mild	
74	43	22/41 (54%)	9/41 (22%)	10/41 (24%)	33/41 (80%)

### The embryos showing the nuclear-localized fluorescence signals have a functional eGFP gene repaired by HDR

**Figure 5:**
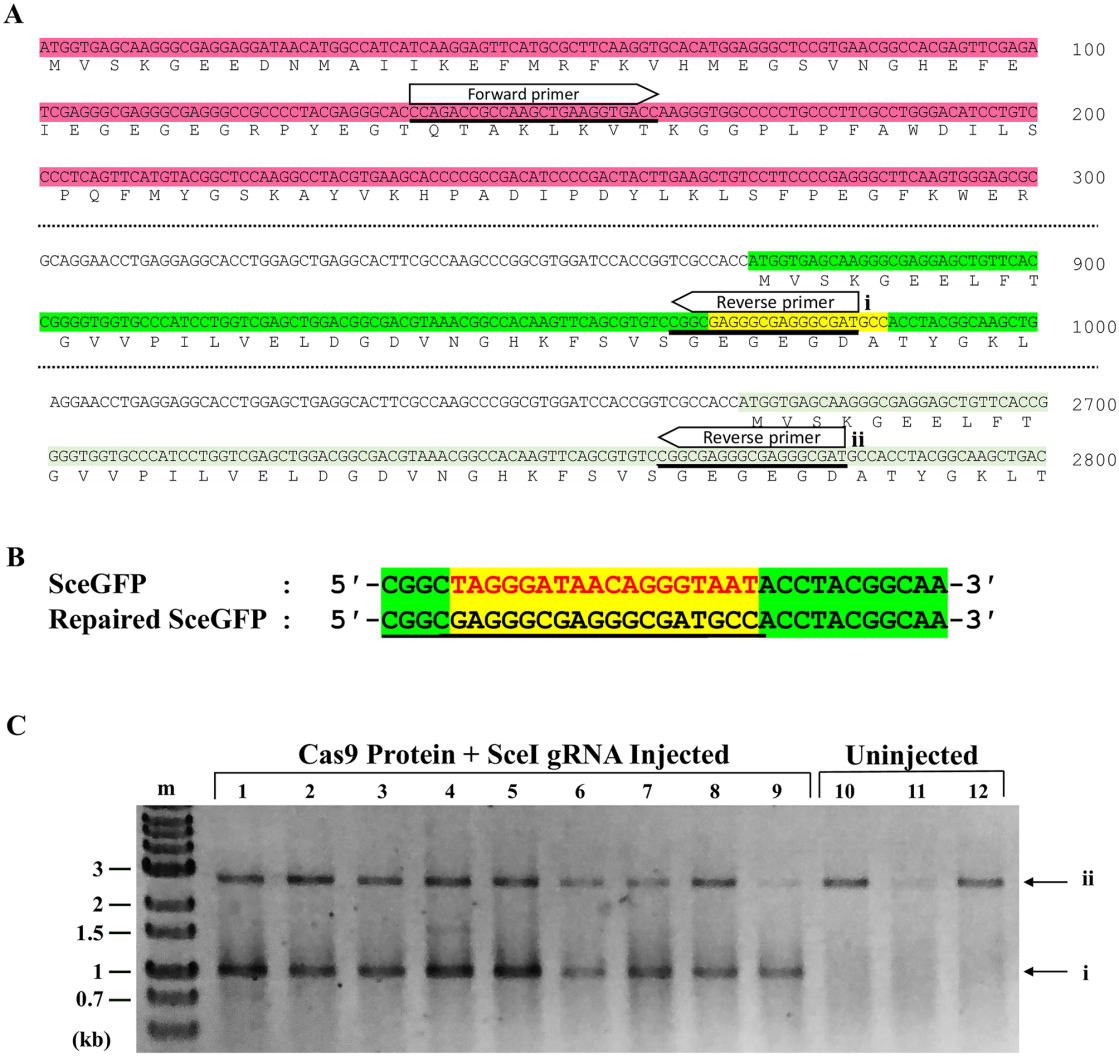
The genotype of DR-GFP transgenic after DSB introduction. (**A**) PCR was performed on uninjected and injected DR-GFP genome using primer pairs indicated by arrows. The forward primer is attached in the mCherry region (pink area) while the reverse primers are attached in two locations, the repaired *SceGFP* (yellow area) and *iGFP* (light green). (**B**) Alignment between *SceGFP* and repaired *SceGFP* or *eGFP*. The reverse primer was designed to bind specifically to the repaired *SceGFP* (underline). (**C**) Gel electrophoresis result. The most left lane indicated the DNA marker (m) followed by amplified genome fragments (lanes 1–9) from Cas9 Protein and SceI gRNA injected embryos. Uninjected DR-GFP (lanes 10–12) was used as the negative control. The primer set amplified the repaired *SceGFP* region with the length 1048 bp (i). The reverse primer was also attached to the *iGFP* region, which resulted in a 2843 bp length PCR product (ii). The full length of the gel is presented in Supplementary Figure S4.

To confirm whether HDR occurred at the genomic level, we extracted genome DNA from uninjected embryos and injected embryos that showed nuclear-localized eGFP fluorescence. PCR was then performed with a forward primer in the *mCherry* region and a reverse primer that recognizes specifically the sequence of the repaired *SceGFP* (Fig. 5A, B). Because the reverse primer also can bind to the *iGFP* sequence that is a template for HDR (Fig. 5A), we expected two bands would appear upon genomic PCR. A higher size band (2,843 bp) was present in all samples, indicating amplification from iGFP sequence (Fig. 5C, ii), while a lower size band (1048 bp) indicating amplification from repaired *SceGFP* sequence was obtained only from embryos injected with Cas9 and SceI gRNA (Fig. 5C, i). These results also suggest the repair of *SceGFP* by Cas9 and SceI gRNA.

**Figure 6:**
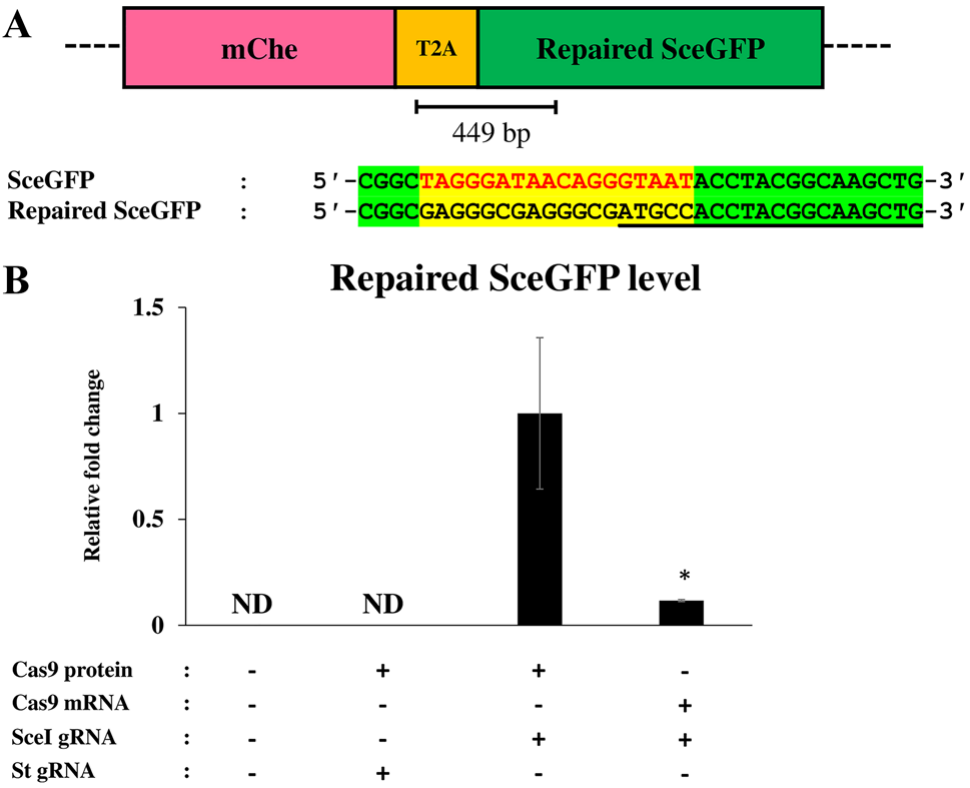
Detection of the functional eGFP transcript by qPCR. (**A**) The position of primers and the region used for quantifying the repaired *SceGFP* level (above) were shown in the black line. The alignment showed that the reverse primer was designed to specifically bind to repaired *SceGFP* fragment (underline). (**B**) Level of repaired *SceGFP* between injected and uninjected samples after the introduction of DSB. The value was quantified by qPCR. There was a significant difference between Cas9 protein and mRNA injection. The values are means and error bars represent standard error (N = 3). *p < 0.05 (Student’s *t-*test). In uninjected embryos and ones injected with Cas9 protein and scarlet gRNA, the repaired SceGFP mRNA was not detected (ND).

We also attempted to develop a qPCR-based method that can detect the repaired SceGFP expression. We designed a forward primer that binds to the T2A-coding sequence of DR-GFP reporter locus, and a reverse primer that specifically binds to repaired *SceGFP* sequence (Fig. 6A). As a model to test this system, we used Cas9-mRNA and Cas9 protein for introducing the DSB on the *SceGFP* because mutagenesis efficiency with Cas9 mRNA was lower than that with Cas9 protein^17^, which suggested Cas9 mRNA induces DSB occurrence to a lesser extent. We introduced the DSB at the *SceGFP* following either optimum condition of Cas9 mRNA (500 ng/µL Cas9 mRNA and 50 ng/μL gRNA) or Cas9 protein injection (1 µM Cas9 protein and 2 μM gRNA)^17,28^. The Dll gRNA was co-injected to evaluate the Cas9 activity in each injection. We confirmed 54% of Cas9 protein injected embryos showed a strong phenotype of second antennae truncation while Cas9 mRNA could only introduce a mild phenotype (Tables 3 and 4). This result implied that Cas9 protein had stronger activity to introduce DSB. Subsequently, the level of repaired *SceGFP* was analyzed using qPCR. By Cas9 protein injection, we observed significantly higher expression of repaired *SceGFP* (~ fivefold) relative to Cas9 mRNA injection. Moreover, neither repaired *SceGFP* signal nor amplification was detected in uninjected embryos as well as scarlet gRNA injected embryos (Fig. 6B, Supplementary Figure S3). Our result shows that qPCR can be used to detect the functional eGFP repaired by HDR.

**Table 4:** Summary of Cas9 mRNA, SceI gRNA, and Dll gRNA co-injection.

Injected	Developed (48hpi)	Truncated antennae			Nuclear-localized eGFP
		Strong	Medium	Mild	
24	10	0	0	8/8 (100%)	Not observed*

*To confirm the integrity of the Cas9 mRNA, the eGFP mRNA was also co-injected for confirmation of the mRNA integrity based on the eGFP fluorescence intensity. This prevented us from observing the nuclear-localized eGFP signals in the Cas9 mRNA-injected embryos.

## Discussion

Here, we successfully integrated the DR-GFP system into *D. magna* genome and visualized HDR occurrence in vivo. We evaluated the functionality of this reporter system by introducing targeted DSB in the reporter site. We observed the eGFP signal and detected PCR products from the repaired eGFP gene in the injected daphniids, demonstrating evidence of detection of HDR in situ based on the eGFP fluorescence. We could also detect the repaired eGFP by qPCR that is potentially used for quantitative measurement of the HDR level following DNA DSB occurrences in the future. Furthermore, ubiquitous expression of mCherry that is bicistronically expressed with the DR-GFP suggests that this reporter system enables us to detect the HDR event in most types of cells.

In this study, we used the DR-GFP line to compare Cas9 mRNA and Cas9 protein ability to introduce DSB and induce HDR in *D. magna*. By using qPCR, we detected significantly higher repaired *SceGFP* expression in the Cas9 protein injected sample (Fig. 6B). This result was in line with a difference in DSB-inducing activity between Cas9 protein and mRNA in *D. magna*^17^. Recently, genome editing techniques using the CRISPR/Cas system are rapidly developing with the emergence of novel and smaller Cas9 proteins, such as *Staphylococcus aureus* derived Cas9 (SaCas9)^35^ and synthetic RNA-guided nuclease (sRGN, surgeons)^36^. As the DR-GFP system was also used to measure HDR DSB and single-strand break (SSB)-inducing activity using Cas9 and Cas9 nickase respectively^34^ in HEK293T cells, we anticipate our DR-GFP *Daphnia* could be a promising tool for the evaluation of new genome editing tools (Fig. 1, genome editors).

The DR-GFP system also has been applied for the screening of genotoxicants such as heavy metals^37^, FDA-approved drugs for cancer therapy^38^ in addition to evaluation of the sensitivity of the cancer cell to gamma-ray irradiation^39^. The aquatic ecosystem is constantly exposed to genotoxicants, and *D. magna* has been long used as a workhorse for ecotoxicology analysis as a biosensor. As the test guideline for acute or chronic toxicity test for *Daphnia* is well established by following the OECD Guideline^40,41^ we believe our DR-GFP *Daphnia* may be suitable for screening genotoxicants in vivo (Fig. 1, genotoxicants).

In recent years, several transgenic animals containing the DR-GFP system have already been established for analyzing the molecular mechanisms of HDR. For instance the generation of DR-GFP reporter mouse for analyzing the HDR frequencies in primary cell types derived from diverse lineages^21^. In *C. elegans*^22^, the DR-GFP system was integrated into the genome to identify a novel role of protein for promoting HDR. *Daphnia* might have a unique DNA repair mechanism to neutralize the genetic drawbacks because its asexual ability might lead to the accumulation of deleterious mutations due to the absence of recombination events via mating. We anticipate the prospect of utilizing this transgenic *Daphnia* for studying the function and roles of HDR in asexual reproduction by manipulating the components of HDR machinery. The result would contribute to a further understanding of evolutionary genomics (Fig. 1, manipulated components).

The HDR efficiency reported in mammals and plants is lower compared to NHEJ^42–44^ because it takes a longer time to complete than NHEJ^42^ and functions only during S and G2 phases when the sister chromatid, the main template to repair DSB, is present^45^. Thus, several approaches have been developed to enhance genome editing by HDR such as inhibiting^46^ or knocking out the key factor of NHEJ^16^, synchronizing and capturing cells at the certain phases^47^, and modifying the Cas9 by fusing it with a key protein necessary in the HDR steps^48^. To evaluate the effects of these approaches on the HDR activity, the reporter system for visualizing the HDR event has been used in mammalian cells^49^. We suggest the potential use of DR-GFP *Daphnia* for optimizing HDR efficiency for instance by impairing the NHEJ repair genes (*Ku70* or *Lig4*) (Fig. 1. manipulated components).

We also acknowledge the limitations of this reporter system. First, in live imaging, it may be difficult to detect the eGFP signals from mutated cells that are located deep inside the tissues, which may lower sensitivity for detecting eGFP positive cells and their quantification. This limitation could be addressed by sorting and counting the eGFP-positive cells using fluorescence-activated cell sorting (FACS). Second, the DR-GFP reporter system can only visualize the presence of HDR events at the reporter locus. This situation may affect the sensitivity for detection of the HDR triggered by environmental genotoxicants or mutagenic agents that may introduce random DSB throughout the genome. Therefore, other approaches to globally visualize HDR events in *Daphnia* may be developed. For instance, fusing the Förster resonance energy transfer (FRET) system in HDR key proteins to provide spatiotemporal visualization of their function^50^. Third, for a comprehensive understanding of the DNA repair mechanism in this species, reporters for detection of the other DNA repair pathways such as NHEJ and SSA need to be developed. This limitation can be addressed by utilizing other reporter systems, such as “traffic light”, a dual fluorescence-based reporter which can visualize HDR and NHEJ repair pathways^51^. However, despite these possible limitations, DR-GFP reporter *Daphnia* would offer a valuable tool for the evaluation of HDR in this ecologically important species.

## Supplementary Information

Supplementary Information.
