# Supplementary material for: Development of transgenic Daphnia magna for visualizing homology-directed repair of DNA
Source: Sci Rep. 2022 Feb 15;12:2497. doi: 10.1038/s41598-022-06526-8 (PMC8847417; doi:10.1038/s41598-022-06526-8)
Supplement: Supplementary file 1 — Supplementary Information. [file 41598_2022_6526_MOESM1_ESM.docx]

**Supplementary information**

**Development of transgenic *Daphnia magna* for visualizing homology-directed repair of DNA**

Rizky Mutiara Fatimah^1,2^, Nikko Adhitama^1^, Yasuhiko Kato^1^, Hajime Watanabe^1^*

^1^ Department of Biotechnology, Graduate School of Engineering, Osaka University, Suita, Osaka, Japan

^2^ Biotechnology Global Human Resource Development Program, Division of Advanced Science and Biotechnology, Department of Biotechnology, Graduate School of Engineering, Osaka University, Suita, Osaka, Japan.

*Corresponding author:

Email: [watanabe@bio.eng.osaka-u.ac.jp](mailto:watanabe@bio.eng.osaka-u.ac.jp)

**Supplementary Figure S1. The full sequence of integrated DR-GFP reporter in the genome.**

**
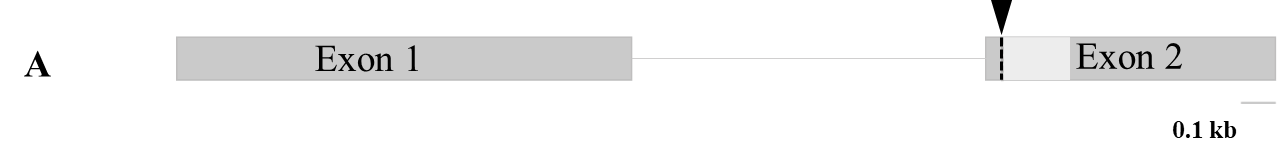
**

**Supplementary Figure S2. Distal-less (Dll) gRNA location.** Illustration of exon 1 and 2 of *Dll* gene in *D*. *magna*. gRNA target (dash line) is located upstream of the homeodomain region (light grey box) of *Dll* gene.

**
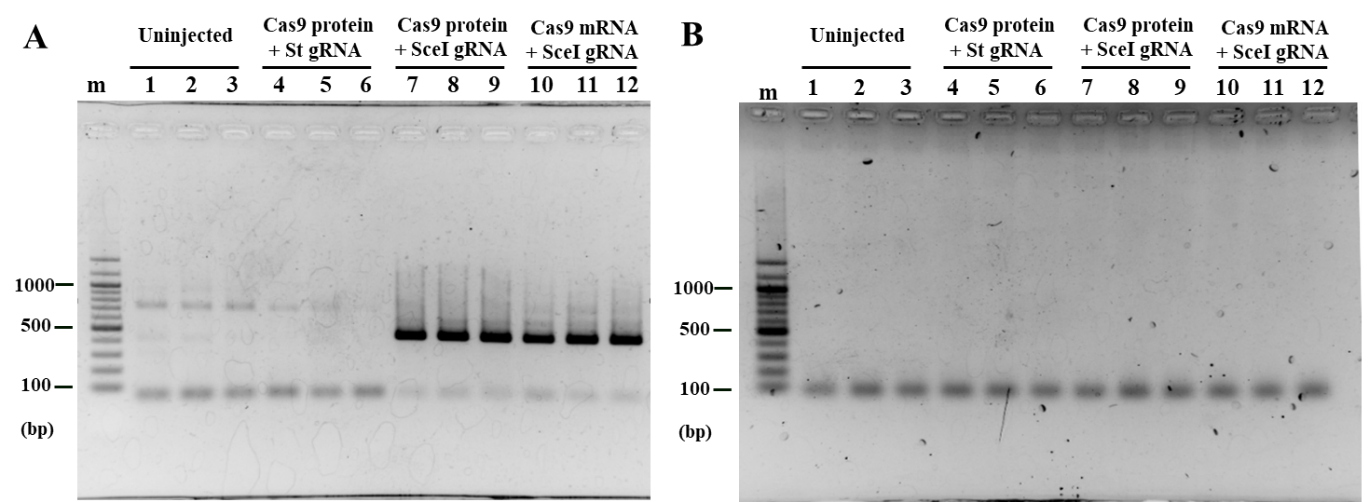
**

**Figure S3. Gel electrophoresis of the qPCR result. (A)** The first lane is the marker (m). Repaired *SceGFP* PCR product which was expected to be 449 bp was only observed in the Cas9 protein (lane 8-10) and mRNA (lane 10-12) co-injection with SceI gRNA. **(B)** The DNA marker was put in the first lane. All of the samples (lane 2-13) showed a band that was expected to be 67 bp that indicated the PCR product of L32 as an endogenous gene for control of cDNA template.

**
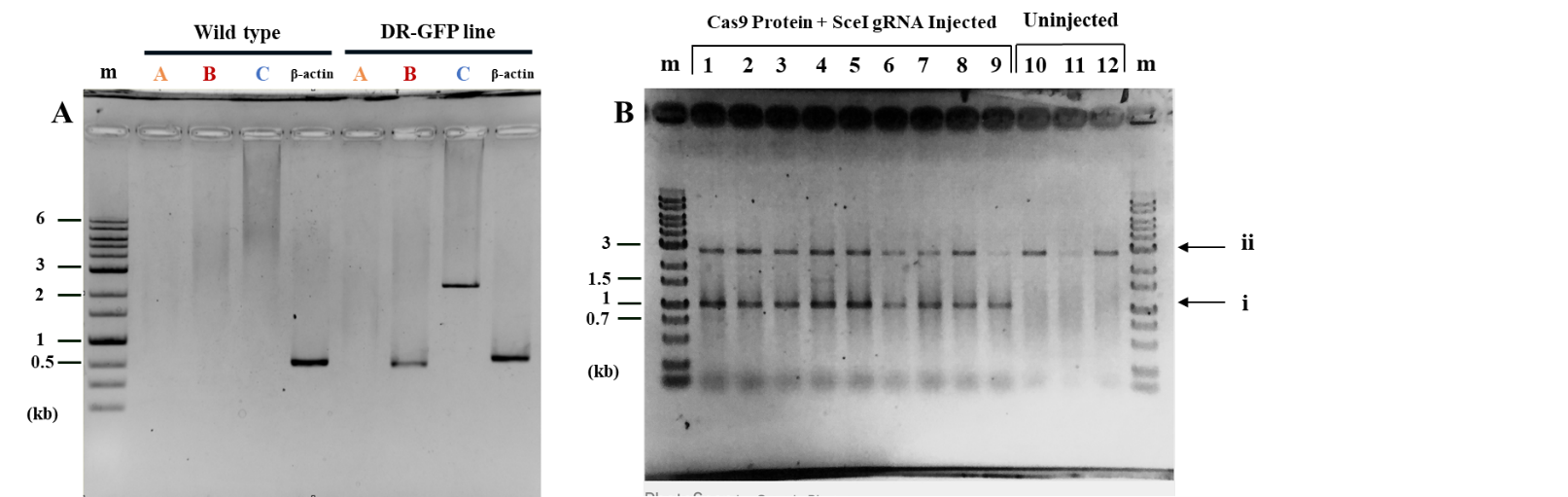
**

**Figure S4. The full length of gels in Figure 3 and Figure 5** **(A)** PCR result for DR-GFP genotyping was visualized by gel electrophoresis. The first lane is the marker (m), followed by fragments A, B, C, and β-actin (beta-actin) for both wild type and DR-GFP line *Daphnia*. In the sample using DR-GFP line *Daphnia,* all fragments except the 5′ junctions, Fragment A, were amplified (lane 6-9). In the wildtype sample only the β-actin band was amplified (line 5). **(B)** PCR result for genotyping after DSB introduction was shown in the gel electrophoresis. The most left lane indicated the DNA marker (m) followed by amplified genome fragments (lanes 1-9) from Cas9 Protein and SceI gRNA injected embryos. Uninjected DR-GFP (lane 10-12) was used as the negative control.  The primer set amplified the repaired *SceGFP* region with the length 1,048 bp (i). The reverse primer was also attached to the *iGFP* region, which resulted in a 2,843 bp length PCR product (ii)

**Supplementary Table S1. Summary of Dll gRNA and Cas9 protein injection**

**(co-injected with St gRNA*)**

| **Injected (16h)** | | 38 |
| --- | --- | --- |
| **Hatch** | | 50% (19/38) |
| **Developed (48h)** | | 68.4% (13/19) |
| **White-eye** | | 84.6% (11/13) |
| **Truncated antennae** | **Strong** | 61.5% (8/13) |
|  | **Medium** | 15.4% (3/13) |
|  | **Mild** | 23.1% (2/13) |

^*^To confirm the functionality of the Cas9 protein, the Dll gRNA was also co-injected together with St gRNA.
